# Carbapenem stewardship with ertapenem and antimicrobial resistance-a scoping review

**DOI:** 10.1590/0037-8682-0413-2020

**Published:** 2020-11-06

**Authors:** Tiago Zequinão, João Paulo Telles, Juliano Gasparetto, Felipe Francisco Tuon

**Affiliations:** 1 Pontifícia Universidade Católica do Paraná, Faculdade de Medicina, Laboratório de Doenças Infecciosas Emergentes, Curitiba, PR, Brasil.; 2 A.C.Camargo Cancer Center, São Paulo, SP, Brasil.

**Keywords:** Antimicrobial stewardship, Ertapenem, Carbapenem-sparing

## Abstract

Consumption of carbapenem has increased due to extended-spectrum beta-lactamase-producing bacteria spreading. Ertapenem has been suggested as a not carbapenem-resistance inducer. We performed a scoping review of carbapenem-sparing stewardship with ertapenem and its impact on the antibiotic resistance of Gram-negative bacilli. We searched PubMed for studies that used ertapenem as a strategy to reduce resistance to carbapenems and included epidemiologic studies with this strategy to evaluate susceptibility patterns to cephalosporins, quinolones, and carbapenems in Gram-negative-bacilli. The search period included only studies in English, up to February 2018. From 1294 articles, 12 studies were included, mostly from the Americas. *Enterobacteriaceae* resistance to quinolones and cephalosporins was evaluated in 6 studies and carbapenem resistance in 4 studies. Group 2 carbapenem (imipenem/meropenem/doripenem) resistance on *A*. *baumannii* was evaluated in 6 studies*.* All studies evaluated *P. aeruginosa* resistance to Group 2 carbapenem. Resistance profiles of *Enterobacteriaceae* and *P. aeruginosa to* Group 2 carbapenems were not associated with ertapenem consumption. The resistance rate of *A. baumannii* to Group 2 carbapenems after ertapenem introduction was not clear due to a lack of studies without bias. In summary, ertapenem as a strategy to spare use of Group 2 carbapenems may be an option to stewardship programs without increasing resistance of *Enterobacteriaceae* and *P. aeruginosa*. More studies are needed to evaluate the influence of ertapenem on *A. baumannii*.

## INTRODUCTION

Ertapenem is a carbapenem with weak activity against *Pseudomonas* spp. and *Acinetobacter* spp*.* ^1^. In randomized controlled trials, ertapenem has been used for severe community-acquired infections and is licensed for intra-abdominal infections, community-acquired pneumonia, skin and soft tissue infections, and complicated urinary infections^2^. The importance of ertapenem increased after dissemination of extended-spectrum β-lactamases (ESBLs), which are now disseminating outside hospitals^3^.

Carbapenems from Group 1 (i.e., ertapenem) and Group 2 (i.e., meropenem) may select for resistant *P. aeruginosa* in vitro^4^. Nevertheless, the selection of carbapenem-resistant *P. aeruginosa* has been shown to be unlikely under physiological ertapenem concentrations. Considering the antimicrobial selective pressure, carbapenem-sparing stewardship strategies have increased in recent years^5^. However, some authors advocate ertapenem as a strategy to reduce resistance to meropenem and imipenem.

Considering the increasing importance of strategies to reduce antibiotic resistance, in this scoping review, we evaluated the effectiveness of an ertapenem-based stewardship strategy in reducing antibiotic resistance in Gram-negative bacilli (GNB).

## METHODS

### Search strategy

Using PubMed, we searched for studies published in English that used ertapenem as a strategy to reduce resistance to any antibiotic. The search included studies from inception to February 2018. The keyword used was “ertapenem” in title and abstract in the advanced search option.

### Data extraction and quality evaluation

Two reviewers (JT and FT) independently screened all studies based on either title or abstract for eligibility. Discrepancies were resolved through discussion. Reviewers then independently extracted the relevant data from all the publications included in the review. A third reviewer evaluated the discrepancies. The methodological quality of each publication was not analyzed using classical scores for randomized clinical trials, but basic elements for an objective evaluation were included in a table for critical analysis.

### Inclusion and exclusion criteria

The inclusion criteria were as follows: *i*) epidemiological studies that compared different periods of ertapenem consumption (i.e.*,* pre vs. post introduction) and *ii*) Evaluation of Group 2 carbapenem susceptibility pattern on Gram-negative bacilli. The exclusion criteria were: *i*) articles classified as case reports or individual data and/or *ii*) undescribed data of ertapenem consumption or susceptibility patterns.

### Definitions and Gram-negative bacilli

The ertapenem consumption model was defined as DDD per patient-day (i.e., DDD/100PD, DDD/1000PD). Susceptibility and resistance evaluation were described in a published original article. Susceptibility patterns were considered according to the Clinical and Laboratory Standards Institute (CLSI) or European Committee on Antimicrobial Susceptibility Testing (EUCAST). The analyzed resistances according to each GNB were: *i*) quinolone in *E. coli* and *K. pneumoniae, ii*) third-generation cephalosporin in *E. coli* and *K. pneumoniae,* and *iii*) carbapenems in *E. coli, K. pneumoniae, A. baumannii, and P. aeruginosa.*

## RESULTS

### Selected articles

**FIGURE 1:**
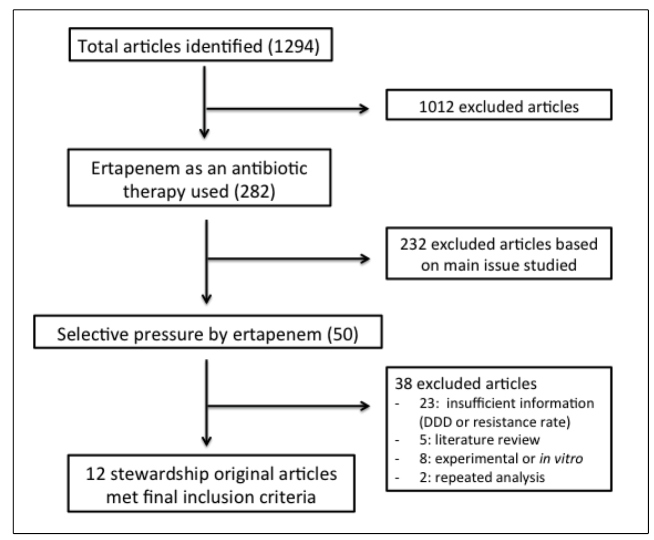
Flowchart for ertapenem studies and antibiotic stewardship.

The search criteria initially identified 1294 articles. After title and abstract reviews, only 12 articles fulfilled the inclusion criteria (Figure 1). The first study was published in 2008 and the last in 2015. The period of analysis varied between 2000 and 2011.

**FIGURE 2:**
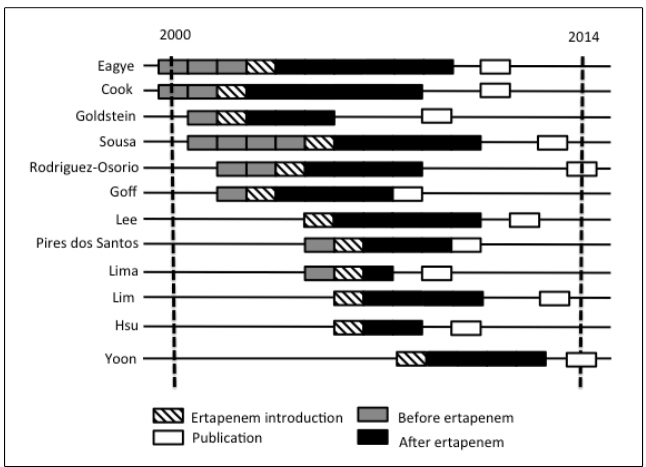
Historical profile of the publications regarding antibiotic stewardship with ertapenem.

Of the articles, 7 were from America^6^ ^-^ ^12^, 4 from Asia^13^ ^-^ ^16^, and 1 from Europe^17^. A timeline of the ertapenem-based stewardship program of each study is presented in Figure 2.

*Enterobacteriaceae* susceptibility patterns to quinolones were evaluated in 5 studies^6^ ^,^ ^11^ ^,^ ^13^ ^-^ ^15^, 6 studies evaluated it susceptibility to cephalosporins^6^ ^,^ ^12^ ^-^ ^16^ *,* and 4 studies to Group 2 carbapenems^6^ ^,^ ^9^ ^,^ ^12^ ^,^ ^13^. Non-fermenting Gram-negative bacilli susceptibility patterns to Group 2 carbapenems were evaluated in 6 studies of *A. baumannii* ^6^ ^,^ ^9^ ^,^ ^13^ ^,^ ^15^ ^-^ ^17^ and all studies evaluated Group 2 carbapenems susceptibility in *P. aeruginosa.*

### Carbapenem consumption

Carbapenem consumption (Groups 1 and 2) was evaluated using different methods. Three studies used the slope curve and nine used comparative periods (before and after consumption). Thus, there was heterogeneity in the metrics used among authors, which complicates the establishment of a median or average value. Only 2 studies demonstrated the substitution tendency of Group 2 carbapenems to ertapenem after its introduction^10^ ^,^ ^11^.

### *E. coli* susceptibility

Three studies analyzed ertapenem consumption and *E. coli* carbapenem resistance rate^6^ ^,^ ^12^ ^,^ ^13^ and one did not specify resistance among *Enterobacteriaceae* isolates^9^ (Table 1 and Supplementary Data - Table 2). Increased ertapenem consumption did not increase *E. coli* resistance to carbapenems. Quinolones were analyzed by 4 studies and third-generation cephalosporins by 6, and presented bias on results^6^ ^,^ ^11^ ^-^ ^16^ (Supplementary Data - Table 2 ). Only 1 publication found a significant increase in quinolone resistance, although higher ciprofloxacin consumption was observed as well^15^. An increased resistance rate to third-generation cephalosporin was observed in 4 studies, but ceftriaxone, ceftazidime, and beta-lactamase inhibitor consumption rates were also higher in 3 studies^6^ ^,^ ^13^ ^,^ ^15^ ^,^ ^16^.

**TABLE 1:** Characteristics of studies included in the review and antibiotics consumption.

Author	Study	Hospital	Antibiotic	Ertapenem	Group 2	Extended-	Fluoroquinolones
(year)	design	settings	consumption	consumption	carbapenem	spectrum	consumption
			measure and metric		consumption	cephalosporins consumption	
Cook et al.(2011)^9^	Retrospective time-series	861 beds medical/surgical	graphic plots DDD/1000 PD ertapenem introduction quarter vs last quarter	0.0 vs 18.0 (p value NP)	10.0 vs 15.00 (p value NP)	20.0 vs 38.0 (p value NP)	90.0 vs 10.0 (p value NP)
Eagye and Nicolau (2011)^8^	Retrospective time-series	25 hospitals	introduction year vs last year (ertapenem) first year vs last year (others) annually DDD/1000 PD	7.27 vs 15.93 (p value NP)	10.39 vs 15.27 (p value NP)	NP	303.84 vs 423.82 (p value NP)
Goff and Mangino (2008)^12^	Retrospective time-seriess	770 beds medical/surgical	first year vs last year annual DDD/1000 PD	3.4 vs 8.9 (RR = 2.61, p<0.001)	IPM 21.5 vs 31.1 (RR=1.45, p<0.001)	CPM 18.8 vs 63.0	NP
Goldstein et al. (2009)^11^	Retrospective interrupted time-series	344 beds	introduction period median vs last period median (ertapenem) post intervention slope (others) monthly DDD/1000 PD	8.0 vs 44.0 (p value NP)	IPM decreased 1.28 (p=0.002)	CPM stable (coefficients NP)	LVX stable (coefficients NP)
Hsu et al. (2010)^15^	Retrospective time-series	4 hospitals totalizing 4000 beds	slope 3 months DDD/1000 PD throughout the entire period	increased 0.079 (p<0.05)	MEM increased 0.057 (p=0.03), IPM decreased 0.057 (p<0.05)	*stable (p=0.23)	** increased 1.677 (p<0.05)
Lee et al. (2013)^13^	Retrospective time-series	1130 beds	slope annually DDD/1000 PD throughout the entire period	increased 4.818 (p<0.001)	MEM increased 1.557 (p<0.001), IPM increased 0.774 (p<0.001)	CRO (p=0.2079), CAZ increased 0.862 (p<0.001), CPM (p=0.544), Cefpirome increased 0.916 (p=0.0426)	CIP increased 0.50 (p<0.001), LVX increased 3.84 (p<0.001), MXF increased 2.674 (p<0.001)
Lim et al. (2013)^14^	Retrospective time-series	NP	first month vs last month DDD/100 PD	0.45 vs 1.2 (p value NP)	MEM 2.0 vs 3.2 (p value NP), IPM 1.8 vs 0.7 (p value NP)	CRO 5.61 vs 12.5 (p value NP), CPM 5.4 vs 4.7 (p value NP)	CIP 1.17 vs 1.3 (p value NP)
Lima et al. (2009)^10^	Retrospective time-series	200 beds trauma/orthopedic	pre period vs post period DDD/1000 PD	0.0 vs 42.6	IPM 46.3 vs 16.1 (p<0.001)	NP	NP
Pires dos Santos et al. (2011)^7^	Retrospective interrupted time-series	749 beds medical/surgical	pre period vs ertapenem period monthly DDD/100 PD	0.05 median throughout ertapenem period	2.6 vs 2.2 (p=0.08)	1.1 vs 0.8 (p<0.05)	10.1 vs 3.6 (p<0.05)
Rodriguez-Osorio et al. (2015)^6^	Retrospective time-series	280 beds medical/surgical	slope 4 months DDD/1000 PD throughout the entire period	increased 15.5 (p<0.001)	† increased 26.6 (p<0.001)	* Decreased 32.2 (p=0.007)	†† decreased 38.6 (p<0.001)
Sousa et al. (2013)^17^	Retrospective interrupted time-series	1445 beds medical/surgical	introduction year vs last year (ertapenem) slope change (others) monthly DDD/100 PD	0.09 vs 2.02 (p<0.001)	stable (p=0.56)	CRO stable (0.082)	stable (p=0.533)
Yoon et al. (2014)^16^	Before-and-after	950 beds medical/surgical	first period vs last period monthly DDD/1000 PD	2.7 vs 7.2 (p<0.001)	20.7 vs 15.5 (p=0.028)	102.2 vs 96.7 (p=0.311)	57.7 vs 67.1 (p=0.102)

**CAZ:** ceftazidime; **CIP:** ciprofloxacin; **CPM:** cefepime; **CRO:** ceftriaxone; **GEN:** gentamicin; **IPM:** imipenem; **LVX:** levofloxacin; **MEM:** meropenem; **MXF:** moxifloxacin; **TZP:** piperacillin/tazobactam; **CR-PA**: carbapenem-resistant *P. aeruginosa*; **NP:** not provided; **OBD:** occupied beds-day; **PD:** patient-day. *CPM, CAZ, and CRO consumption. **CIP, LVX, and MXF consumption. † MEM and IPM consumption. †† CIP and ofloxacin consumption.

### *K. pneumoniae* susceptibility

Three studies analyzed ertapenem consumption and *K. pneumoniae* carbapenem resistance rate^6^ ^,^ ^12^ ^,^ ^13^, and one did not specify resistance among *Enterobacteriaceae* isolates^9^(Table 1). Increased consumption of ertapenem changed the susceptibility patterns of carbapenems in some studies. One study showed a slight improvement in carbapenem susceptibility^13^. Another study found a higher incidence of resistance to Group 2 carbapenems on univariate analysis; however, higher consumption of meropenem/imipenem was observed^6^. Quinolones and third-generation cephalosporin susceptibility were analyzed in 4 and 6 studies respectively^6^ ^,^ ^12^ ^-^ ^16^ (Supplementary Data - Table 2). Increased third-generation cephalosporin resistance was observed in 4 studies^6^ ^,^ ^12^ ^,^ ^13^ ^,^ ^16^.

### *A. baumannii* susceptibility

Six studies analyzed ertapenem consumption and *A. baumannii* carbapenem resistance rates^6^ ^,^ ^9^ ^,^ ^13^ ^,^ ^15^ ^-^ ^17^ (Table 1 and Supplementary Data - Table 2). Increased consumption was associated with a decrease in susceptibility patterns in 2 studies^13^ ^,^ ^15^. Nevertheless, both of them increased meropenem and/or imipenem consumption and 1 increased resistance only on univariate analysis^13^ ^,^ ^15^.

### *P. aeruginosa* susceptibility

Twelve studies analyzed ertapenem consumption and *P. aeruginosa* carbapenem resistance rates (Table 1 and Supplementary Data - Table 2)^6^ ^-^ ^17^. Results were variable. Three studies demonstrated significant susceptibility pattern improvement^9^ ^,^ ^11^ ^,^ ^17^. Six did not observe significant changes in resistance patterns^7^ ^,^ ^8^ ^,^ ^10^ ^,^ ^12^ ^,^ ^15^ ^,^ ^16^. Three studies demonstrated a higher carbapenem resistance rate after ertapenem introduction^6^ ^,^ ^13^ ^,^ ^14^. However, 2 studies increased Group 2 carbapenem consumption as well^6^ ^,^ ^14^, and one of them did not present significant statistical results on multivariate analysis^6^.

## DISCUSSION

We conducted a scoping review to better understand Gram-negative bacilli antibiotic resistance and ertapenem consumption. Twelve studies evaluated ertapenem consumption as an intervention to change Group 2 carbapenem resistance. After this strategy, the Group 2 carbapenem was reduced in 3 studies. Carbapenem resistance in *Enterobacteriaceae* did not increase after ertapenem consumption. However, non-fermenting Gram-negative bacilli demonstrated changes in susceptibility patterns. Carbapenem-resistant in *A*. *baumannii* increased in 2 of 6 studies, while 4 observed no difference. *P. aeruginosa* improved carbapenem susceptibility in 3 of the 12 studies, while 7 observed no differences and 2 increased carbapenem resistance.

The hypothesis that ertapenem has the potential to select *P. aeruginosa* and *A. baumannii* resistant to Group 2 carbapenems is due to its limited action on non-fermenting Gram-negative bacilli (NF-GNB). Previous reviews did not observe higher rates of carbapenem resistance in NF-GNB despite an increase in ertapenem consumption^18^ ^,^ ^19^.

*The* carbapenem resistance rate in *E. coli* did not increase after ertapenem consumption. Studies have observed changes in *E. coli* susceptibility only to cephalosporins and quinolones*.* Hsu et al. (2010) observed that increased resistance to ceftriaxone and ciprofloxacin correlated with increasing consumption^15^. Lee et al. (2010) found increased susceptibility to ceftazidime and levofloxacin in addition to increasing its consumption^13^.

*K. pneumoniae* carbapenem resistance rate did not increase overall and it was positively affected by routine utilization of ertapenem in one study. Lee et al. (2010) observed an improvement in susceptibility to carbapenems, ceftazidime, and levofloxacin after ertapenem introduction^13^. Changes in the resistance rate of *K. pneumoniae* to cephalosporin and quinolones were observed. Hsu et al. (2010) demonstrated lower resistance to ceftriaxone and ciprofloxacin but this was not correlated with antibiotic consumption^15^. Goff and Mangino (2008) observed higher resistance to cephalosporins in the latter period and inferred it was due to multiple hospitalizations^12^. Overall, *Enterobacteriaceae* carbapenem resistance was not affected by ertapenem consumption. These results are in accordance with stable CRE colonization rates after patients using ertapenem as surgical prophylaxis^20^.

*A. baumannii* demonstrated predominantly no difference in the results and worst susceptibility patterns in 2 studies^13^ ^,^ ^15^. However, there was a significant increase in consumption in Group 2 carbapenems and other broad-spectrum antibiotics. Yoon et al converged with these results when they concluded that carbapenem resistance rate is correlated with Group 2 carbapenem consumption^16^.

Carbapenem-resistant *P. aeruginosa* was not increased by ertapenem use in the majority of studies. Increased resistance rates were demonstrated in a study with higher Group 2 carbapenem consumption^13^. Nevertheless, Lim et al. (2013) observed a negative impact on carbapenem susceptibility even with no difference in Group 2 carbapenem consumption in both periods^14^. Similar to *A. baumannii*, other studies found that *P. aeruginosa* resistance was affected by Group 2 carbapenem consumption but not by ertapenem^21^ ^,^ ^22^. These studies converged with two positive results in the present review^11^ ^,^ ^17^, in which lower resistance was correlated with less usage of imipenem. Only one study directly associated ertapenem consumption with better carbapenem susceptibility^9^.

The present study has several limitations. Methods heterogeneity may make certain conclusions difficult when studies were not comparable between each other. Other factors may have influenced the carbapenem resistance rate of Group 2, such as higher meropenem/imipenem consumption, without multivariate analysis evaluation. However, this article presents a relevant issue in infectious disease practice and may help stewardship programs to adequately choose carbapenem therapeutic regimens without affecting the bacterial resistance rate.

## CONCLUSION

The majority of studies did not demonstrate a rising Group 2 carbapenem resistance rate in *Enterobacteriaceae* and *P*. *aeruginosa* after ertapenem introduction. The rate of resistance to Group 2 carbapenems on *A*. *baumannii* is not clear. However, studies did demonstrate that worsening carbapenem resistance was associated with Group 2. If a carbapenem group is needed in an antimicrobial stewardship program, ertapenem may be an option to spare Group 2 carbapenem usage without increasing resistance in *Enterobacteriaceae* and *P. aeruginosa*.
