# Supplementary material for: Carbapenem stewardship with ertapenem and antimicrobial resistance-a scoping review
Source: Rev Soc Bras Med Trop. 2020 Nov 6;53:e20200413. doi: 10.1590/0037-8682-0413-2020 (PMC7670755; doi:10.1590/0037-8682-0413-2020)
Supplement: Supplementary file 1 [file 1678-9849-rsbmt-53-e20200413-suppl1.pdf]

SUPPLEMENTARY DATA - TABLE 2: Microorganism resistance to antibiotics and conclusions.

	Microorganisms analysis measure and metric	Impact on NF-GBN resistance to Group 2 carbapenems	Impact on Enterobacteriaceae resistance to group 2 carbapenems	Impact on Enterobacteriaceae resistance to cephalosporins/quinolones	Correlation between ertapenem consumption and GNB resistance to carbapenems	Comments	Conclusion
Cook et al. (2011)[9]	graphic plots % of resistants	- *P. aeruginosa* 24% vs 16% (p value NP) - *A. baumannii* no difference (p value NP)	no difference (p value NP)	NP	- *P. aeruginosa* % of resistants correlation coefficient = -0.45815, p=0.003 - *A. baumannii* NP	There was a correlation of ciprofloxacin use with percentage and rate of carbapenems resistant *P. aeruginosa*	*P. aeruginosa*: decreased resistance to carbapenems *A. baumannii*: no difference *E. coli*: no difference *K. pneumoniae*: no difference
Eagye and Nicolau (2011)[8]	first year vs last year % of susceptible	- *P. aeruginosa* 85.4% vs 81.0% (p=0.99) - *A. baumannii* NP	NP	NP	NP	*P. aeruginosa* susceptibility was not associated with ertapenem use neither other antibiotic classes across the study	*P. aeruginosa*: no difference *A. baumannii*: NP *E. coli*: NP *K. pneumoniae*: NP
Goff and Mangino (2008)[12]	first year vs last year annual % of susceptible	- *P. aeruginosa* to IPM 71% vs 72% (p=0.92) - *A. baumannii* NP	- *E. coli* to IPM: 100% vs 100% (p value NP) - *K. pneumoniae* to IPM: 99% vs 99% (p value NP)	- *E. coli* ESBL: 1,07% vs 2.00% (p=0.30) - *K. pneumoniae* ESBL: 4% vs 18% (p<0.001)	NP	*K. pneumoniae* ESBL isolates increased was justified due to community or transplanted patients with multiple hospitalization on later period	*P. aeuruginosa*: no difference *A. baumannii*: NP *E. coli:* no difference *K. pneumoniae*: no difference
Goldstein et al. (2009)[11]	slope monthly % of susceptibles	- *P. aeruginosa* increased 1.74 (p<0.001) - *A. baumannii* NP	NP	-*E. coli* to LVX: 90% vs 83% (p value NP) - *K. pneumoniae*: NP	NP	The author associated improved susceptibilities to IPM decreasing consumption	*P. aeuruginosa*: increased susceptibility to Group 2 carbapenems *A. baumannii*: NP *E. coli*: NP *K. pneumoniae*: NP
Hsu et al.(2010)[15]	slope 3 months resistants/1000 PD	- *P. aeruginosa* to IPM stable (p=0.37) - *A. baumannii* to IPM increased on blood isolates (p=0.03)	NP	-*E. coli* to CRO, CIP: increased 0.032 (p<0.05), increased 0.031 (p=0.02) respectively. - *K. pneumoniae* to CRO, CIP: decreased 0.074 (p<0.05), decreased 0.091 (p<0.05) respectively.	- *P. aeruginosa* no significant correlation.- *A. baumannii* positive correlation (R2=0.394) on IPM resistance	*A. baumannii* resistance to carbapenems was also correlated with LEV and TZP consumption. *E. coli* resistance was also correlated with quinolones, TZP and CRO consumption. *K. pneumoniae* resistance was not correlated with antibiotic consumption.	*P. aeuruginosa*: no difference *A. baumannii*: Increased resistance to Group 2 carbapenems on blood isolates *E. coli*: NP *K. pneumonia*: NP
Lee et al. (2013)[13]	slope annual % of susceptible	- *P. aeruginosa* to MEM and IPM: decreased 0.798 (p=0.0184) and stable (p=0.1786) -*A. baumannii* to MEM and IPM: decreased 4.136 (p=0.007) and	-*E. coli* to MEM and IPM: stable (p=0.9209 and p=1.000) -*K. pneumoniae* to MEM and IPM: increased 1.058 (p<0.001) and stable (p=0.7877)	-*E. coli* to CAZ, CIP, LVX: increased 8.903 (p<0.001), stable (p=0.2822), increased 17.020 (p=0.0021) respectively. - *K. pneumoniae* to CAZ, CIP, LEV: increased 11.619 (p<0.0027), stable (p=0.6844), increased 20.722 (p=0.0023).	- *P. aeruginosa* to MEM and IMI: correlation coefficient = -0.148, p=0.0330 and correlation coefficient = -0.355, p=0.1731 - *A. baumannii* to MEM and IMI: correlation coefficient = -0.796, p<0.001 and correlation coefficient = -1.077, p<0.001	There was a significant negative correlation of ertapenem use and MEM susceptibility on GNB, but the same with MEM use and MEM susceptibility. There was a significant increase in *E. coli* susceptibility to CAZ, but in other hand, total *E. coli*	*P. aeuruginosa*: decreased susceptibility to Group 2 carbapenems *A. baumannii*: decreased susceptibility to Group 2 carbapenems *E. coli*: no difference *K. pneumoniae*: increased susceptibility to Group 2 carbapenems

		decreased<br>5.195<br>(p<0.001)				ESBL-producing<br>increased.	
Lim et al. (2013)[14]	first month vs last month resistants/1000 PD	- *P. aeruginosa* 0.25 vs 0.35 (p value NP)<br>- *A. baumannii* NP	NP	-*E. coli* to CRO, CIP: 1.6 vs 2.0 (p value NP), 3.2 vs 3.7 (p value NP) respectively.<br>- *K. pneumoniae* to CRO, CIP:2.4 vs 1.5 (p value NP), 2.2 vs 1.1 (p value NP) respectively.	- *P. aeruginosa* correlation coefficient = 0.5648, R2=0.3190, p=0.089<br>-*A. baumannii* correlation coefficient = -0.6485, R2=0.0911, p=0.397	Conclusions were based on correlation of ertapenem use and incidence rate of resistant pathogens.<br>There was correlation between ertapenem increasing use and cefepime decreasing. MEM also increased its usage but was not statistically measured.	*P. aeuruginosa*: increased resistance to Group 2 carbapenems<br>*A. baumannii*: no difference<br>*E. coli:* Group 2 carbapenems NP<br>*K. pneumoniae*: Group 2 carbapenems NP
Lima et al. (2009)[10]	pre period vs post period<br>% of resistants	- *P. aeruginosa* 20.0% vs 0.0% (p>0.05)<br>- *A. baumannii* NP	NP	NP	NP	Although a noticed difference in resistance proportions, these numbers are about 20 vs 18 strains, and no difference was noticed in the trend over time	*P. aeuruginosa*: no difference<br>*A. baumannii*: NP<br>*E. coli*: NP<br>*K. pneumoniae*: NP
Pires dos Santos et al.(2011)[7]	pre period vs ertapenem period resistants/1000 PD	- *P. aeruginosa*0.51 vs 0.43 (p=0.33)<br>- *A. baumannii* NP	NP	NP	NP	Introduction of ertapenem was associated with a decrease in IPM and MEM use. By multivariate analysis, only alcohol hand-gel was correlated with the decrease in CR-PA in the last period	*P. aeuruginosa*: no difference<br>*A. baumannii*: NP<br>*E. coli*: NP<br>*K. pneumoniae*: NP
Rodriguez-Osorio et al. (2015)[6]	slope<br>4 months resistants/1000 isolates	- *P. aeruginosa* increased 6.26 (p<0.05)<br>- *A. baumannii* *i*ncreased 25.39 (p<0.001)	- *E. coli* increased 0.46 (p<0.05)<br>- *K. pneumoniae* increased 8.06 (p<0.001)	-*E. coli* to CAZ, CRO, CIP: increased 6.92 (p<0.001), increased 10.00 (p<0.001), decreased 1.45 (p>0.05) respectively.<br>- *K. pneumoniae* to CAZ, CRO, CIP: increased 11.72 (p<0.001), 17.52 (p<0.001), 2.29 (p>0.05) respectively.	NP	In a multiple linear regression analysis adjusted for length of stay, hospital acquired infections and other 10 antibiotic usage ertapenem was not associated with changes in resistances	*P. aeuruginosa*: no difference<br>*A. baumannii*: no difference<br>*E. coli*: no difference<br>*K. pneumoniae*: no difference
Sousa et al. (2013)[17]	slope<br>monthly resistants/1000 isolates on ertapenem period	-*P. aeruginosa* to IPM decreased 0.005 (p<0.001)<br>-*A. baumannii* to IPM stable (p=0.54)	NP	NP	Correlation was not calculated between ertapenem use and incidence of IPM resistant strains.<br>However, decreased IPM consumption was correlated to decreased IPM resistance	In a multiple regression analysis CIP, GEN, IPM, outbreaks and other variables were associated with the incidence density of IPM resistance strains.	*P. aeuruginosa*: decreased resistance to Group 2 carbapenems<br>*A. baumannii*: no difference<br>*E. coli*: NP<br>*K. pneumoniae*: NP
Yoon et al. (2014)[16]	first period vs last period monthly<br>% resistants	- *P. aeruginosa* 18.1% vs 19.4% (p=0.648)<br>- *A. baumannii* 52.2% vs 69.9% (p<0.001)	NP	- *E. coli* ESBL 31.8% vs 43.4% (p<0.001)<br>- *K. pneumoniae* ESBL: 20.1% vs 41.7% (p<0.001)	There was a correlation between Group 2 carbapenem consumption during a previous month and carbapenem resistant *A. baumannii* proportion on following month (p=0.03)	Despite an increased proportion of carbapenem resistant *A. baumannii,* there was no correlation with ertapenem consumption on previous month and increased proportion on following month (p=0.941)	*P. aeuruginosa*: no difference<br>*A. baumannii*: no difference<br>*E. coli*: NP<br>*K. pneumoniae*: NP

CAZ: ceftazidime, **CIP:** ciprofloxacin, **CPM:** cefepime, **CRO:** ceftriaxone, **GEN:** gentamicin, **IPM:** imipenem, **LVX:** levofloxacin, **MEM:** meropenem, **MXF:** moxifloxacin, T**ZP:** piperacillin/tazobactam, **CR-PA:** carbapenem-resistant *P. aeruginosa*, **NP:** Not provided, **OBD:** occupied beds-day, **PD:** patient-day.
